# Blocking autophagosome closure manifests the roles of mammalian Atg8-family proteins in phagophore formation and expansion during nutrient starvation

**DOI:** 10.1080/15548627.2024.2443300

**Published:** 2024-12-18

**Authors:** Van Bui, Xinwen Liang, Yansheng Ye, William Giang, Fang Tian, Yoshinori Takahashi, Hong-Gang Wang

**Affiliations:** a Division of Pediatric Hematology and Oncology, Department of Pediatrics, The Pennsylvania State University College of Medicine, Hershey, PA, USA; b Department of Biochemistry and Molecular Biology, The Pennsylvania State University College of Medicine, Hershey, PA, USA; c Advanced Light Microscopy Core Facility, The Pennsylvania State University College of Medicine, Hershey, PA, USA; d Department of Pharmacology, The Pennsylvania State University College of Medicine, Hershey, PA, USA

**Keywords:** Autophagosome closure, ESCRT, LIR, membrane expansion, phagophore formation, The mammalian Atg8 family of proteins

## Abstract

Macroautophagy/autophagy, an evolutionarily conserved cellular degradation pathway, involves phagophores that sequester cytoplasmic constituents and mature into autophagosomes for subsequent lysosomal delivery. The *ATG8* gene family, comprising the *MAP1LC3/LC3* and *GABARAP/GBR* subfamilies in mammals, encodes ubiquitin-like proteins that are conjugated to phagophore membranes during autophagosome biogenesis. A central question in the field is how Atg8-family proteins are precisely involved in autophagosome formation, which remains controversial and challenging, at least in part due to the short lifespan of phagophores. In this study, we depleted the autophagosome closure regulator VPS37A to arrest autophagy at the vesicle completion step and determined the roles of mammalian Atg8-family proteins (mATG8s) in nutrient starvation-induced autophagosome biogenesis. Our investigation revealed that *LC3* loss hinders phagophore formation, while *GBR* loss impedes both phagophore formation and expansion. The defect in membrane expansion by *GBR* loss appears to be attributed to compromised recruitment of ATG proteins containing an LC3-interacting region (LIR), including ULK1 and ATG3. Moreover, a combined deficiency of both *LC3* and *GBR* subfamilies nearly completely inhibits phagophore formation, highlighting their redundant regulation of this process. Consequently, cells lacking all *mATG8* members exhibit defects in downstream events such as ESCRT recruitment and autophagic flux. Collectively, these findings underscore the critical roles of mammalian Atg8-family proteins in phagophore formation and expansion during autophagy.**Abbreviation**: AIM: Atg8-family interacting motif; ADS: Atg8-interacting motif docking site; ATG: autophagy related; BafA1: bafilomycin A_1_; CL: control; ESCRT: endosomal sorting complex required for transport; FACS: fluorescence activated cell sorting; GBR: GABARAP; GBRL1: GABARAPL1; GBRL2: GABARAPL2; GBRL3: GABARAPL3; HKO: hexa-knockout; IP: immunoprecipitation; KO: knockout; LDS: LC3-interacting-region docking site; LIR: LC3-interacting region; mATG8: mammalian Atg8-family protein; MIL: membrane-impermeable ligands; MPL: membrane-permeable ligands; RT: room temperature; Stv: starved; TKO: triple-knockout; TMR: tetramethylrhodamine; UEVL: ubiquitin E2 variant-like; WCLs: whole cell lysates; WT: wild-type.

## Introduction

Macroautophagy, hereafter referred to as autophagy, is a highly conserved cellular process responsible for the degradation and recycling of proteins and organelles [1]. The process begins with the formation of a cup-shaped membrane structure, known as the phagophore, which expands and eventually seals to sequester cytoplasmic materials by forming a double-membrane vesicle called the autophagosome. The autophagosome then fuses with the lysosome to deliver sequestered cargoes for hydrolytic degradation. Autophagy is activated in response to various stresses, such as nutrient starvation, to maintain cell homeostasis. Dysregulated autophagy has been linked to various human diseases including cancer, neurodegenerative disorders, metabolic diseases, and infectious diseases [2].

The molecular understanding of autophagosome biogenesis has been advanced by the studies on ATG (autophagy related) proteins [3,4]. Based on their functions in autophagy, ATG proteins can be classified into the following groups: the Atg1/ULK kinase complex, the Atg9-containing vesicles, the phosphatidylinositol 3-kinase (PtdIns3K) complex, the Atg2-Atg18/WIPI complex, and the Atg8-family protein conjugation system. The Atg8-family proteins are ubiquitin-like proteins that are covalently conjugated to both the outer and inner leaflet of phagophores. Through the two hydrophobic pockets that form an LC3-interacting region (LIR) or an Atg8-family interacting motif (AIM) docking site (LDS or ADS, respectively), they interact with other ATG proteins during autophagy [5]. In yeast, which carries only one *ATG8* gene, its deletion impairs autophagosome generation, while the amount of Atg8 protein has been shown to determine the size of autophagosomes [6,7]. In mammalian cells, there are a total of eight Atg8 homologs (mATG8s), which can be segregated into two subfamilies: the LC3 (MAP1LC3A/LC3A, MAP1LC3B/LC3B, MAP1LC3B2/LC3B2, MAP1LC3C/LC3C) and GABARAP (GABARAP/GBR, GABARAPL1/GBRL1, GABARAPL2/GBRL2, GABARAPL3/GBRL3) subfamilies [8]. Among them, LC3B2 is expressed only in specific tissues, primarily in the brain and testes, whereas *GBRL3* is considered a pseudogene [9,10]. Similar to the yeast Atg8 studies [6,7], earlier studies in mammalian cells suggest the critical roles of mATG8s and their conjugation in phagophore formation or growth [11–14]. However, recent studies have shown that nearly complete autophagosome-like structures can form in the absence of mATG8s and their conjugation machineries. The autophagic structures detected in these studies are unclosed or permeant (having holes) [15–17], incapable of fusing with lysosomes [18,19] or defective in undergoing efficient inner membrane degradation upon lysosome fusion [20]. Thus, the later studies have shifted our perspective, suggesting that mammalian Atg8-family proteins primarily influence late rather than early stages of autophagy [8]. The discrepancies between yeast and mammalian systems, as well as inconsistencies between earlier and recent studies in mammalian cells, remain unclear but may be attributed to the short lifespan of phagophores [21] and the absence of a tool to dissect Atg8-family protein’s roles in autophagosome biogenesis. In addition to their involvement in autophagosome formation and maturation, mATG8s play critical roles in selective cargo sequestration by recruiting LIR-containing autophagy receptors, including SQSTM1/p62 [22].

The endosomal sorting complex required for transport (ESCRT) is a membrane remodeling machinery that drives membrane scission away from the cytoplasm [23]. We and others have previously found that ESCRT mediates autophagosome completion by sealing the phagophore and separating the inner and outer autophagosomal membranes [24–28]. Although ESCRT mediates various other cellular membrane scission processes, including endosomal protein sorting, cytokinesis, nuclear envelope reformation, and repairs of plasma membrane and lysosomes [23], its role in mammalian autophagosome closure is uniquely regulated by the N-terminus of the ESCRT-I subunit VPS37A, which contains a ubiquitin E2 variant-like (UEVL) domain flanked by two unstructured regions [24,29]. Interestingly, a recent study has shown that the N-terminal unstructured region of VPS37A contains a putative LIR, which promotes ESCRT translocation during autophagy by interacting with a subset of mATG8s, specifically LC3A and GBR [17]. Moreover, while plants lack a *VPS37A* homolog, they encode the plant-unique ESCRT component *FREE1*, which contains a classical AIM motif required for interaction with ATG8 and autophagosome closure [30]. These studies suggest a role of mATG8s in ESCRT recruitment during autophagy. However, unlike ATG8 conjugation-defective plants [30], the loss of Atg8-family proteins in mammalian cells does not lead to the accumulation of phagophores [17,18]. Thus, the specific roles of mammalian Atg8-family proteins in ESCRT targeting during autophagy remain elusive.

In this study, we generated knockouts (KOs) of *LC3s* (*LC3A, LC3B, LC3*), *GBRs* (*GBR, GBRL1, GBRL2*), and both subfamilies (hexa-knockout, HKO) in U-2 OS cells, and explored the effects of their loss on starvation-induced autophagy. We found that Atg8-family proteins regulate autophagic flux independently of their reported roles in ESCRT targeting. By further depleting VPS37A to inhibit autophagosome biogenesis at the vesicle completion step, we revealed the critical roles of mATG8s in controlling the number and size of phagophores during autophagy. Additionally, our analysis of *LC3* and *GBR* triple KO (TKO) cells demonstrated that phagophore formation is redundantly regulated by both subfamilies, while membrane expansion is primary controlled by the GBR family. These results emphasize the importance of mATG8-dependent phagophore biogenesis in regulating autophagic flux in mammalian cells.

## Results

### mATG8-deficient U-2 OS cells are defective in starvation-induced autophagy

**Figure 1.**
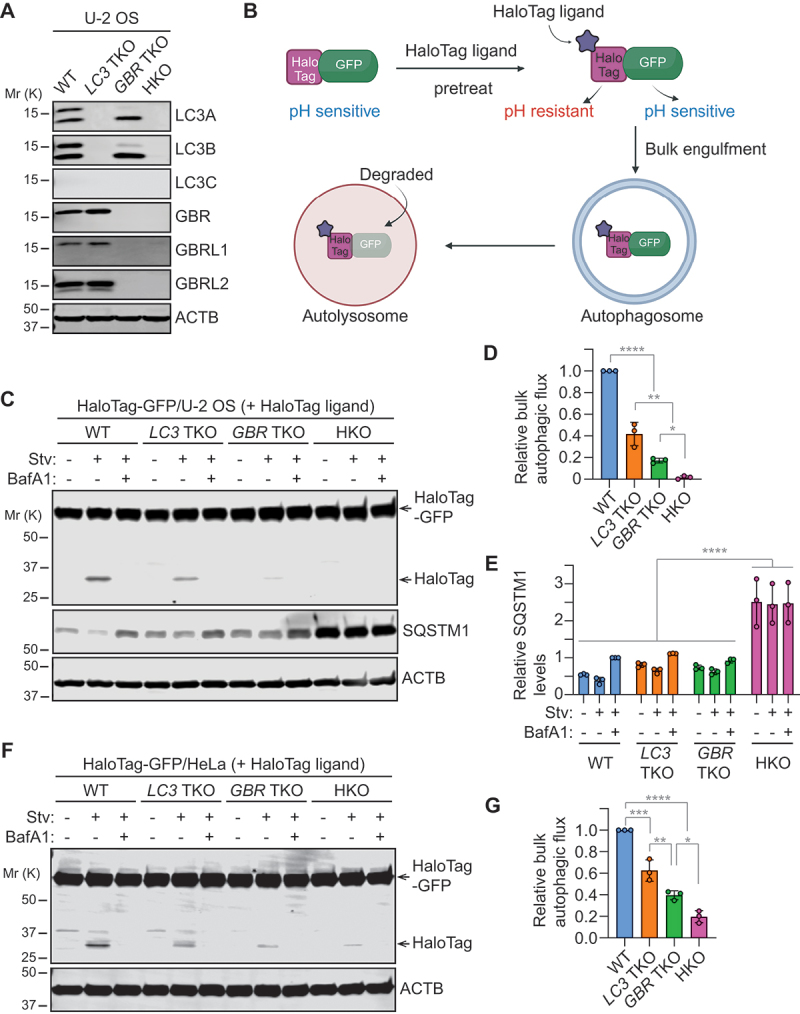
mATG8 proteins are crucial for both bulk and selective autophagy during nutrient starvation. (A) Western blot analysis validating the absence of targeted Atg8-family proteins in *LC3* triple-knockout (TKO), *GABARAP* (*GBR*) TKO, and *mATG8* hexa-knockout (HKO) U-2 OS cells. To maximize the amount of mATG8 proteins, cells were starved in the presence of 100 nM bafilomycin A_1_ (BafA1) for 3 h. (B) schematic diagram of the HaloTag-GFP reporter-based bulk autophagy flux assay (created in BioRender. Bui, V. (2024) BioRender.com/t38p691). (C-G) Western blot analysis of the indicated U-2 OS (C-E) and HeLa (F and G) cells that were stably transduced with the *HaloTag-GFP* reporter, pulse-labeled for 20 min with tetramethylrhodamine (TMR)-conjugated membrane-permeable HaloTag ligand (MPL), and starved in the presence or absence of 100 nM BafA1 for 6 h. (D and G) quantification of HaloTag/(HaloTag-GFP + HaloTag) ratio relative to WT cells under starvation conditions in C and F (*n* = 3). (D) quantification of SQSTM1 levels relative to WT cells under starvation and BafA1 for 6 h (*n* = 3). All values in D, E and G are presented as mean ± SD. One-way ANOVA test was performed followed by Tukey’s multiple comparison test. The *p*-values in D: ****p < 0.0001, **p = 0.003, *p = 0.0363; in E: ****p < 0.0001; and in F: ****p < 0.0001, ***p = 0.0003, **p = 0.0072, *p = 0.016.

To study the role of mammalian Atg8-family (mATG8) proteins in autophagosome biogenesis, we employed the CRISPR-Cas9 genome editing system to generate triple-knockouts (TKOs) of *LC3* (*LC3A, LC3B, LC3*), *GBR* (*GBR, GBRL1, GBRL2*), and both subfamilies (hexa-knockout, HKO) in U-2 OS human osteosarcoma cells. We selected U-2 OS cells because their dependence on ESCRT for autophagosome closure has been well-established in previous studies [24,25,27,28,31]. This characteristic enables us to specifically investigate phagophore formation and expansion by arresting autophagosome biogenesis at the final step. Successful depletion of each mATG8 protein was verified by immunoblotting (Figure 1A). Additionally, as LC3C protein expression was undetectable in U-2 OS cells, disruption of the *LC3C* gene was further confirmed by Sanger sequencing (Fig. S1). To determine if the depletion of all six members is sufficient to inhibit autophagy in U-2 OS cells, the resultant cells were subjected to autophagic flux analysis using the HaloTag-GFP bulk autophagy reporter [32]. HaloTag is a modified haloalkane dehalogenase designed to covalently bind to synthetic HaloTag ligands [33]. Upon expression of HaloTag fused with GFP, cytoplasmic proteins are non-selectively engulfed by autophagosomes and delivered to lysosomes, where proteolytically released HaloTag and GFP are degraded (Figure 1B). When the reporter-expressing cells are pre-incubated with HaloTag ligands, the ligand-bound HaloTag, but not GFP, becomes resistant to pH-sensitive proteolysis in the lysosome. Thus, by monitoring the free, ligand-bound HaloTag level, bulk autophagic flux can be measured. As expected, in ligand-preincubated wild-type (WT) cells, nutrient starvation increased the level of released HaloTag in a manner dependent on the lysosomal acidification inhibitor bafilomycin A_1_ (BafA1) (Figure 1C). In contrast, starvation-induced HaloTag release was nearly completely suppressed in HKO cells, indicating a defect in bulk autophagic flux due to the loss of the targeted *mATG8* members (Figure 1C,D). Moreover, consistent with the previous study on *mATG8* HKO HeLa cells [18], the lysosomal turnover of the autophagy receptor SQSTM1/p62 was also impaired in HKO U-2 OS cells (Figure 1C,E). We also observed a significant reduction in the lysosomal turnover of both released HaloTag and SQSTM1 in TKO cells (Figure 1C–E). Notably, the inhibitory effect of *GBR* TKO was more pronounced than that of *LC3* TKO, while the highest level of suppression was detected in HKO cells. Similar trends were observed in *mATG8* TKO and HKO HeLa cells, which were previously generated by Lazarou’s group [18] (Figure 1F,G), although the inhibitory effect of starvation-induced HaloTag release by *mATG8* HKO was milder than that in U-2 OS cells (Figure 1D,G). Collectively, we established *mATG8*-deficient U-2 OS cells that are defective in both bulk and selective autophagy, highlighting the non-redundant and redundant functions of mATG8 family members in regulating autophagic flux during starvation.

### mATG8 loss abrogates ESCRT targeting to SQSTM1/p62 bodies during autophagy

**Figure 2.**
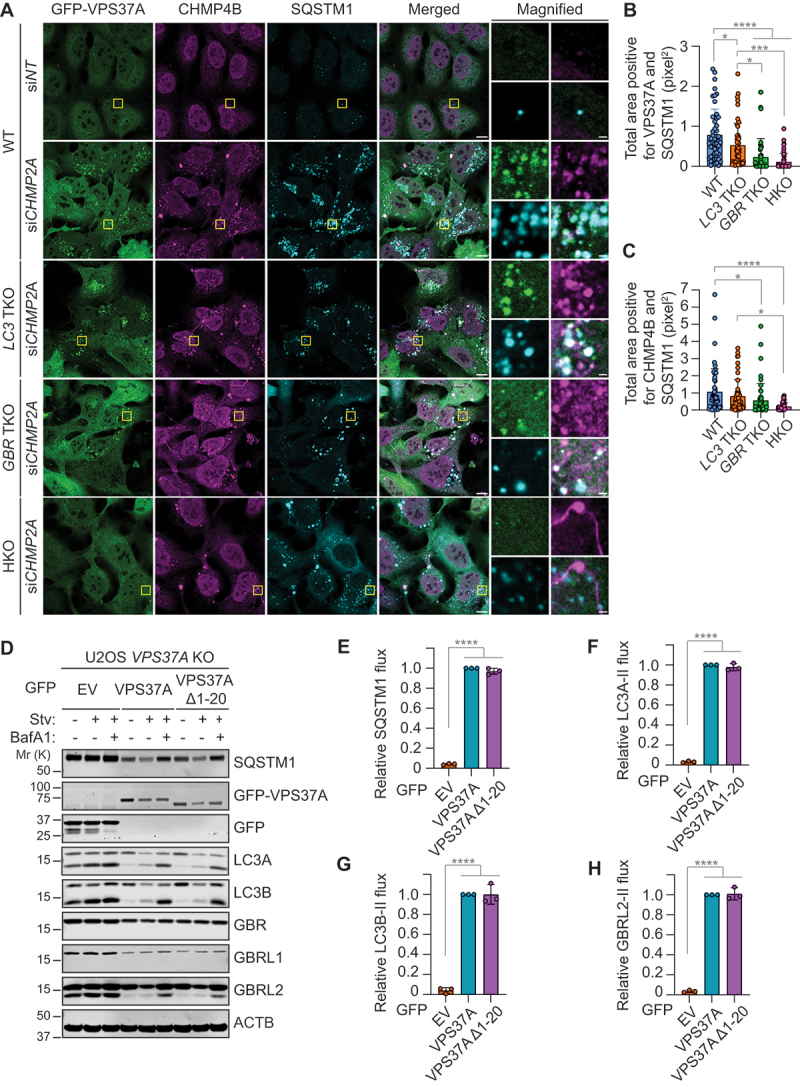
*mATG8* loss impairs ESCRT recruitment to SQSTM1/p62 bodies. (A) confocal images of GFP-VPS37A-expressing WT, *LC3* TKO, *GBR* TKO, and HKO U-2 OS cells that were transfected with si*NT* or si*CHMP2A* for 40 h, starved for 3 h, and stained for CHMP4B and SQSTM1. Scale bars: 10 μm; 1 μm in magnified images. (B and C) quantification of total area per cell positive for SQSTM1 and VPS37A (B) and CHMP4B (C) in A (*n* = 50 cells). (D) Western blot analysis of *VPS37A* KO U-2 OS cells that were stably transduced with either *GFP-VPS37A*, *GFP-VPS37A Δ1–20* or control *GFP*-empty vector (EV), and starved in the presence or absence of 100 nM BafA1 for 3 h. (E-H) quantification of (“Stv+BafA1”-’Stv’)/“Stv+BafA1” ratio relative to GFP-VPS37A-expressing *VPS37A* KO U-2OS cells in D (*n* = 3) for starvation-induced fluxes of SQSTM1 in E and lipidated forms of LC3A-II (F), LC3B-II (G), and GBRL2-II (H). All values in B, C, E, F, G, H are presented as mean ± SD. One-way ANOVA test was performed followed by Tukey’s multiple comparison test. The *p*-values in B: ****p < 0.0001, ***p = 0.0002, *p = 0.0447 and 0.0164 (from left to right); in C: ****p < 0.0001, *p = 0.0474 and 0.011 (from left to right); in E: ****p < 0.0001; in F: ****p < 0.0001; in G: ****p < 0.0001; and in H: ****p < 0.0001.

Inhibition of VPS37A-mediated autophagosome closure severely impairs autophagic flux [24]. mATG8 family members, specifically LC3A and GBR, reportedly interact and recruit VPS37A to autophagosomal membranes [17]. Therefore, we examined the effect of *mATG8* loss on VPS37A targeting during autophagy. To enhance the detection of phagophore-targeted VPS37A, the experiment was performed under conditions of ESCRT-III *CHMP2A* depletion, which inhibits the VPS4-dependent disassembly of ESCRT and autophagosome closure. Consistent with our previous study [24], accumulation of VPS37A signals was detected at SQSTM1-labeled autophagosome formation sites (SQSTM1 bodies) [34] in *CHMP2A*-depleted WT U-2 OS cells (Figure 2A,B). These VPS37A and SQSTM1-double-positive structures were also positive for the ESCRT-III subunit CHMP4B (Figure 2A,C), confirming successful ESCRT stabilization. However, the accumulation of VPS37A and CHMP4B at SQSTM1 bodies upon *CHMP2A* depletion was significantly reduced by the loss of either *LC3* or *GABARAP* subfamily members, and further diminished by the loss of both, implying a role of mATG8 proteins in ESCRT targeting [17]. Notably, the inhibitory effect of *mATG8* depletion on ESCRT recruitment appeared to be confined to autophagosome closure, as CHMP4B accumulation on VPS37A-negative structures persisted in HKO cells following *CHMP2A* depletion. To assess the functional significance of mATG8-dependent VPS37A recruitment in autophagic flux, we introduced the mATG8 binding-defective VPS37A mutant (Δ1–20) [17] into *VPS37A* KO U-2 OS cells. Consistent with the dispensability of the VPS37A N-terminus for ESCRT-I complex formation [24,35], the mutation did not disrupt the interaction of VPS37A with the ESCRT-I subunit TSG101 (Fig. S2A-E). Unexpectedly, however, we did not detect an interaction of VPS37A with any mATG8 members, regardless of the mutation (Fig. S2A-E). Moreover, the mutant VPS37A reduced the basal levels of SQSTM1, LC3A-II, LC3B-II, and GBRL2-II, and restored their starvation-induced lysosomal turnover similarly to wild-type VPS37A (Figure 2D–H). Interestingly, GBR and GBRL1 migrated as single bands at their respective molecular weights, and their levels were unaffected by VPS37A depletion or BafA1 treatment, suggesting their rapid lipid deconjugation during starvation-induced autophagosome formation. Collectively, these findings indicate that the reported VPS37A-mATG8 interaction [17] is dispensable for starvation-induced autophagy and suggest that that the mATG8 family may play critical roles in autophagosome biogenesis upstream of autophagosome closure in U-2 OS cells.

### mATG8 proteins play a crucial role in phagophore formation and expansion

**Figure 3.**
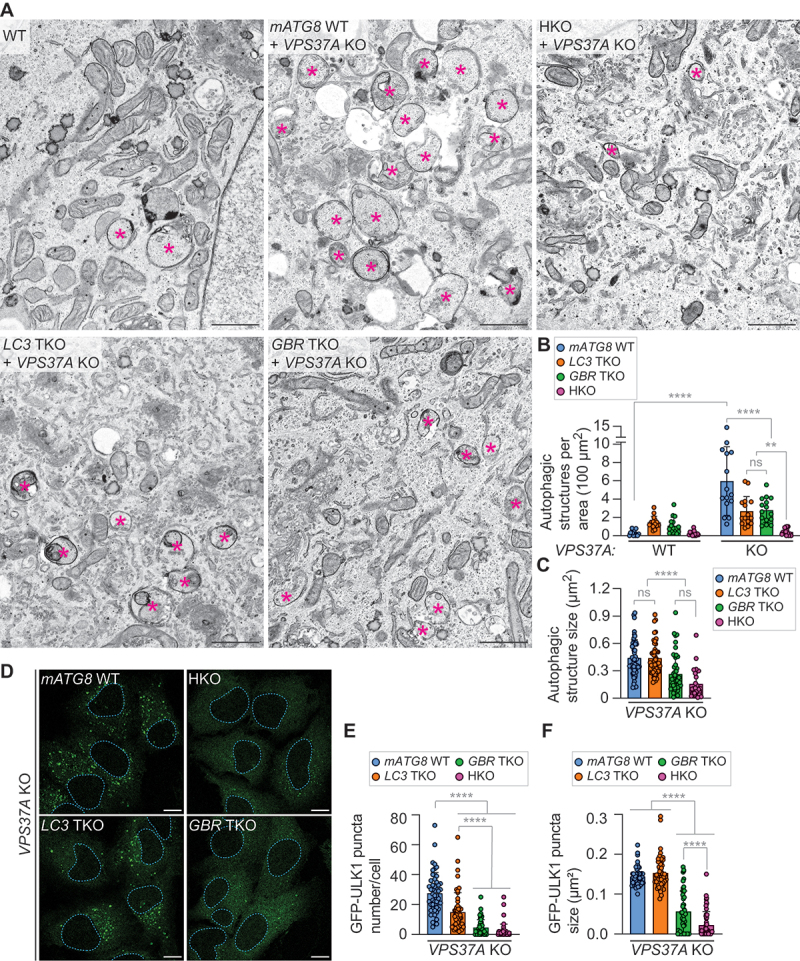
mATG8 proteins are crucial for phagophore formation and expansion. (A) electron microscopy (EM) images of WT, *VPS37A* KO (mATG8 WT+*VPS37A* KO), *VPS37A*-deficient *LC3* TKO (*LC3* TKO+*VPS37A* KO), *VPS37A*-deficient *GBR* TKO (*GBR* TKO+*VPS37A* KO), and *VPS37A*-deficient *mATG8* HKO (HKO+*VPS37A* KO) U-2 OS cells that were starved for 3 h. Asterisks indicate immature autophagic structures including phagophores. Scale bars: 1 μm. (B and C) quantification of the number of autophagic structures per cytoplasmic area (B; *n* = 15 cells) and the size of autophagic structures (C; *n* = 28 structures for HKO, 50 structures for the remaining groups) in A. (D) confocal images of the indicated GFP-ULK1-expressing U-2 OS cells that were starved for 3 h. Scale bars: 10 μm. (E and F) quantification of the number (E) and mean size (F) of GFP-ULK1 puncta per cell in D (*n* = 50 cells). All values in B, C, E and F are presented as mean ± SD. One-way ANOVA test was performed followed by Tukey’s multiple comparison test. The *p*-values in B: ****p < 0.0001, **p = 0.0047; in C: ****p < 0.0001; in E: ****p < 0.0001; and in F: ****p < 0.0001; ns, not significant.

To examine the roles of mATG8 proteins in autophagosome formation, we took advantage of *VPS37A* KO to arrest autophagosome biogenesis at the closure step [24]. As amphisome (endosome-autophagosome hybrid)-like structures accumulate in *mATG8*-deficient cells [17], but not in *VPS37A*-deficient cells [24], this approach allows to minimize the complications of endosome fusion and more precisely assess the roles of mATG8 in phagophore expansion. After confirming successful VPS37A depletion in both *mATG8* TKO and HKO U-2 OS cells by immunoblotting (Fig. S3), the resultant cells were starved to induce autophagy and subjected to electron microscopy. In the presence of all six mATG8 members, we observed an accumulation of immature autophagic structures upon *VPS37A* loss (WT vs “*mATG8* WT; *VPS37A* KO” in Figure 3A,B). These structures were comparable in size to those detected in WT cells (Figure 3A), supporting that ESCRT inhibition impairs autophagosome closure without affecting membrane growth [24,25]. In contrast, *VPS37A*-deficient HKO (HKO; *VPS37A* KO) cells exhibited significantly smaller and fewer autophagic structures compared to *VPS37A*-deficient, *mATG8*-intact cells (*mATG8* WT; *VPS37A* KO). Notably, while both *LC3* and *GRB* subfamily losses led to a partial decrease in the number of autophagic structures (Figure 3B), only GBR subfamily loss exhibited a significant reduction in the size of autophagic structures (Figure 3C). These results are in line with the observation that *GBR* TKO cells displayed a greater inhibitory effect on autophagic flux than *LC3* TKO cells (Figure 1C–E). To further assess the impact of *mATG8* loss on autophagosome biogenesis, we transduced *VPS37A*-deficient TKO and HKO cells with *GFP-ULK1* and analyzed them using confocal microscopy. ULK1, a component of the autophagy-initiation kinase complex, translocates to autophagosome formation sites to promote phagophore formation but dissociates from the membrane upon autophagosome closure [24,36]. We detected a strong reduction in the number and size of GFP-ULK1-positive structures in *VPS37A*-deficient *GBR* TKO and HKO cells (Figure 3D–F). *LC3* TKO cells also showed a significant reduction in ULK1-positive structures, although the effect was less pronounced compared to *GBR* TKO and HKO cells, and their sizes were comparable to those in *VPS37A-*deficient, *mATG8*-WT cells. These results align with the electron microscopy data (Figure 3A–C) and suggest that while both the LC3 and GBR subfamilies contribute to phagophore formation, phagophore expansion is primarily regulated by the GBR subfamily members in U-2 OS cells. Overall, by inhibiting autophagosome closure, we successfully demonstrated the critical roles of mATG8 proteins in phagophore biogenesis.

### Ectopic expression of an individual mATG8 member can restore autophagy in cells lacking all six members of the mATG8 family

**Figure 4.**
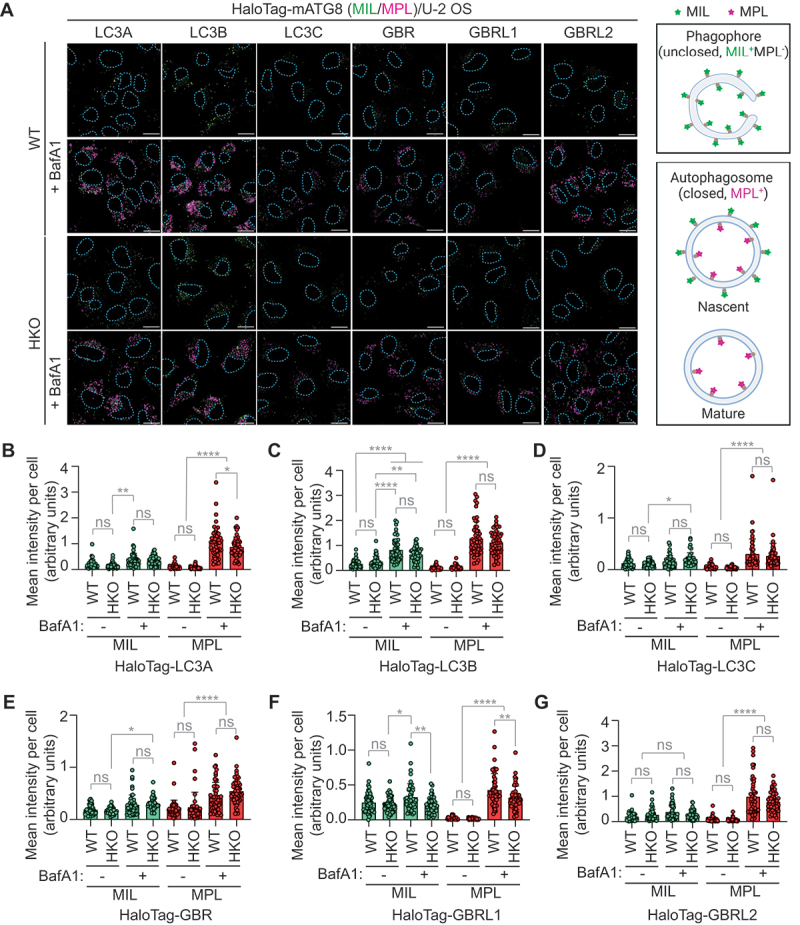
Overexpression of an Atg8-family member can compensate for the loss of other family members and restore autophagy in HKO cells. (A) confocal images of WT and HKO U2-OS cells that were stably transduced with *HaloTag-LC3A*, *LC3B*, *LC3C*, *GBR*, *GBRL1*, and *GBRL2*, starved in the presence or absence of 100 nM BafA1 for 3 h, and subjected to the HaloTag-mATG8 autophagosome completion assay with Alexa Fluor 488-conjugated MIL (green) and tetramethylrhodamine-conjugated MPL (magenta). Scale bars: 10 μm. Expected unclosed (phagophores, MIL^+^ MPL^−^) and closed (nascent autophagosomes, MIL^+^ MPL^+^; mature autophagosomes, MIL^−^ MPL^+^) structures distinguishably labeled by the assay are illustrated on the right (created in BioRender. Bui, V. (2024) BioRender.com/q66q604). (B-G) quantification of cytoplasmic fluorescence intensities of MIL and MPL in each cell in figure 4A (*n* = 50 cells). The mean fluorescence intensity, normalized to the cytoplasmic area, is shown. All values in B-G are presented as mean ± SD. One-way ANOVA test was performed followed by Tukey’s multiple comparison test. The *p*-values in B: ****p < 0.0001, **p = 0.0094, *p = 0.045; in C: ****p < 0.0001, **p = 0.0026; in D: ****p < 0.0001, *p = 0.0401; in E: ****p < 0.0001, *p = 0.0171; in F: ****p < 0.0001, **p = 0.0019 and 0.0038 (from left to right), *p = 0.0146; and in G: ****p < 0.0001; ns, not significant.

Our results suggest that the LC3 and GBR subfamilies may have redundant and non-redundant functions in phagophore formation and expansion. To investigate the roles of individual mATG8 members in autophagosome formation, we fused each *mATG8* member with *HaloTag* and transduced them into *mATG8* HKO and control WT cells (Fig. S4B). These cells were then starved in the presence or absence of BafA1 and subjected to the HaloTag-mATG8 autophagosome completion assay (previously referred to as the HaloTag-LC3 autophagosome completion assay) (Figure 4A). This assay uses membrane-impermeable ligands (MIL) and membrane-permeable ligands (MPL) conjugated with two different fluorescent molecules to distinguish unsealed (MIL^+^ MPL^−^) phagophores from sealed (MPL^+^) autophagosomes (Fig. S4A) [25]. In WT cells, the levels of MIL^+^ and MPL^+^ signal intensities varied among the reporters (with the highest signals observed for HaloTag-LC3A, LC3B, and GBRL2). In the presence of BafA1, a significant MPL^+^ signal increase was detected for each reporter (Figure 4A–G), indicating that all the reporters translocated to phagophores can be sequestered upon closure and subjected to lysosomal degradation. Surprisingly, despite the substantial reduction in the number of autophagic structures in both *LC3* and *GBR* TKO cells (Figure 3A,B), comparable levels of MPL^+^ signal increases were detected in both WT and HaloTag-mATG8-expressing HKO cells upon BafA1 treatment (Figure 4A–G). These MPL^+^ signal increases reflected lysosomal turnover of phagophore-conjugated HaloTag-mATG8, as evidenced by the increases in the lipidated forms of the reporters by BafA1 (Fig. S4C and D). Notably, unlike autophagosome closure-defective *VPS37A* KO cells [24], the levels of MIL^+^ structures in HaloTag-mATG8-expressing HKO cells were comparable to those in WT cells. Similar results were obtained in *mATG8* HKO HeLa cells using HaloTag-LC3B and HaloTag-GBRL2 (Fig. S4E-G). Collectively, these results suggest that upon ectopic expression, each mATG8 member can not only rescue the phagophore formation defect but also restore autophagosome completion and subsequent maturation in HKO cells.

**Figure 5.**
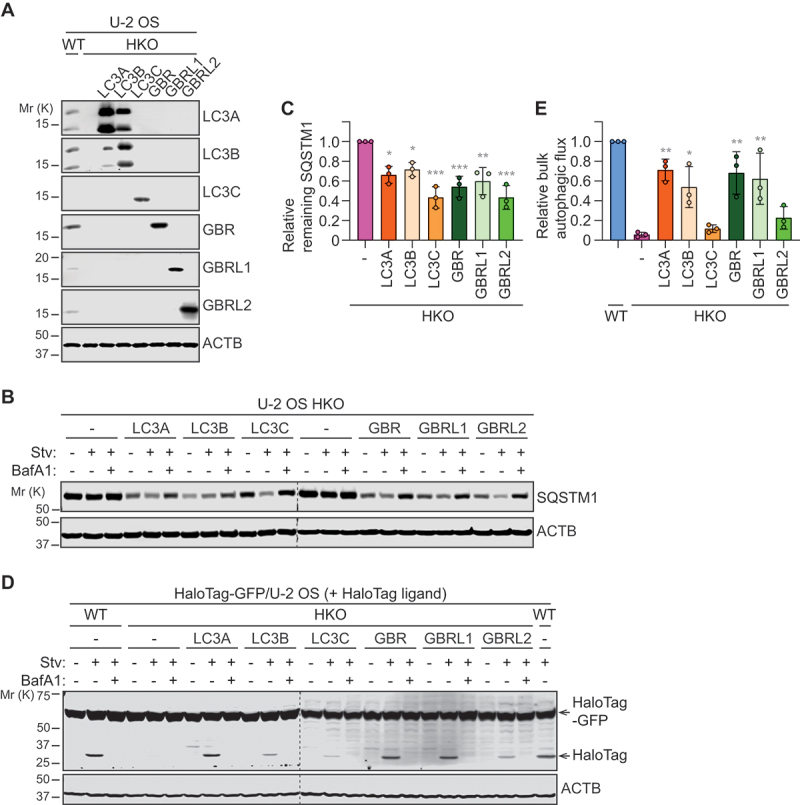
Overexpression of an Atg8-family member can restore autophagy in HKO cells. (A) Western blot analysis of wild-type and *mATG8* HKO U-2 OS cells stably transduced with individual *mATG8* family members). Note that LC3A and LC3B antibodies may exhibit cross-reactivity. (B) Western blot analysis of HKO U-2 OS cells that were transduced with individual non-tagged *mATG8* members and starved in the presence or absence of 100 nM BafA1 for 3 h. (C) quantification of “Stv”/’Stv+BafA1’ ratio for remaining SQSTM1 after starvation relative to HKO cells in B (*n* = 3). (D) Western blot analysis of the indicated U-2 OS cells that were transduced with *HaloTag-GFP*, pulse-labeled with TMR for 20 min, and starved in the presence or absence of 100 nM BafA1 for 6 h. (E) quantification of HaloTag/(HaloTag-GFP + HaloTag) ratio relative to WT cells under starvation conditions in D (*n* = 3). All values in C and E are presented as mean ± SD. One-way ANOVA test was performed followed by Tukey’s multiple comparison test. The *p*-values in C: ***p = 0.0001, 0.0009, 0.0001 (from left to right), **p = 0.0032, *p = 0.0129, 0.0442 (from left to right); and in E: **p = 0.0015, 0.0024, 0.0059 (from left to right), *p = 0.0222.

Next, we determined if the restoration of autophagosome formation by ectopically expressing each mATG8 member rescues the substrate degradation defect in HKO cells. As HaloTag-fused mATG8 proteins were found to be defective in SQSTM1 turnover (data not shown) similar to GFP-tagged mATG8 proteins for mitophagy [18], the assays were performed by transducing HKO cells with non-tagged mATG8s (Figure 5A). We found that exogenous expression of each member promoted SQSTM1 turnover (Figure 5B,C) and HaloTag-GFP reporter processing in HKO cells (Figure 5 5D,E), indicating the restoration of both selective and bulk autophagic flux. Interestingly, unlike the autophagosome completion assay data (Figure 4A–G), some members, specifically LC3C and GBRL2, only partially restored HaloTag-GFP processing. This discrepancy could be attributed to differences in assay sensitivity or additional roles of the mATG8 members in functional autolysosome formation.

### The LDS motif in mATG8 is crucial for autophagic flux but not essential for autophagosome closure

**Figure 6.**
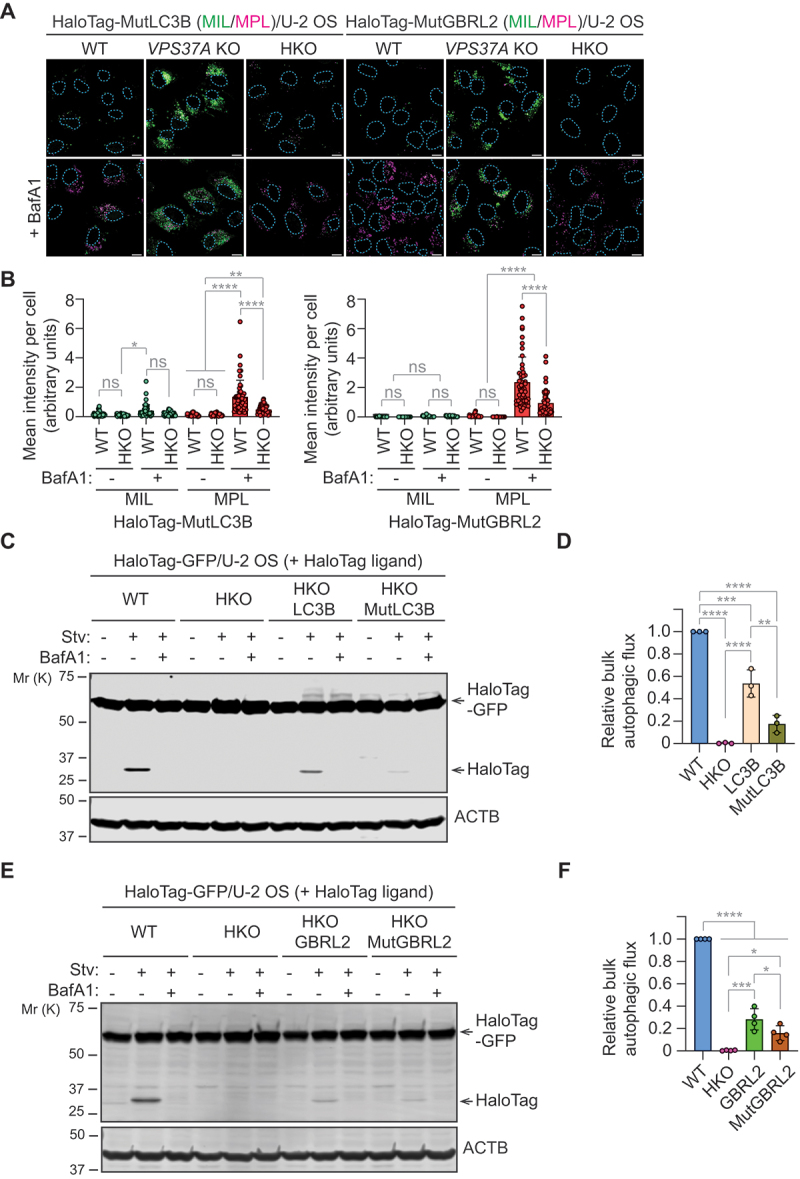
The LIR motif-docking sites (LDSs) of mATG8 proteins are important for autophagosome biogenesis. (A) confocal images of WT, *VPS37A* KO, and *mATG8* HKO U-2 OS cells that were stably transduced with *HaloTag-LC3B (K51A; MutLC3B)* and *HaloTag-GBRL2 (Y49A, L50A; MutGBRL2)*, starved in the presence or absence of 100 nM BafA1 for 3 h, and subjected to the HaloTag-mATG8 autophagosome completion assay with Alexa Fluor 488-conjugated MIL (green) and tetramethylrhodamine-conjugated MPL (magenta). Scale bars: 10 μm. (B) quantification of cytoplasmic fluorescence intensities of MIL and MPL in each cell in A (*n* = 50 cells). The mean fluorescence intensity, normalized to the cytoplasmic area, is shown. (C-F) Western blot analysis of HaloTag-GFP-expressing WT and HKO U-2 OS cells that were stably transduced with non-tagged wild-type and mutant forms of *LC3B* (C) and *GBRL2* (E), pulse-labeled for 20 min with TMR, and starved in the presence or absence of 100 nM BafA1 for 6 h. Quantification of HaloTag/(HaloTag-GFP + HaloTag) ratio relative to WT cells under starvation conditions in C and E are shown in D and F, respectively. All values in B, D and F are presented as mean ± SD. One-way ANOVA test was performed followed by Tukey’s multiple comparison test. The *p*-values in B: ****p < 0.0001, **p = 0.0015, *p = 0.0172; in D: ****p < 0.0001, ***p = 0.0002, **p = 0.0012; and in F: ****p < 0.0001, ***p = 0.0001, *p = 0.0129 and 0.0482 (from top to bottom); ns, not significant.

Through the LC3-interacting region (LIR)-docking site (LDS), mATG8 proteins interact with various regulators involved in autophagosome formation, in addition to autophagic cargo molecules [5,17,30]. Although the N-terminal LIR-like motif of VPS37A [17] is dispensable for autophagy (Figure 2D–H), mATG8 proteins may regulate autophagosome closure by recruiting other LIR-containing proteins, such as ATG2A or unidentified ESCRT molecules [14,30]. To explore this possibility, we generated LIR binding-defective mutant forms of mATG8 (LC3B^K51A^; GBRL2^Y49A,L50A^) [37] by introducing point mutations into the *HaloTag-mATG8* constructs. The successful disruption of LIR binding by the mutations was verified through co-immunoprecipitation analysis with the LIR-containing autophagy receptor SQSTM1 (Fig. S5A and B), followed by monitoring its lysosomal turnover during starvation (Fig. S5C-F). The HaloTag-mATG8 assay results showed that overexpression of the mutations did not significantly affect phagophore formation and autophagosome closure in the presence of endogenous mATG8 proteins, as demonstrated by the accumulation of MIL+ phagophores and MPL+ autophagosomes in *VPS37A*-deficient and BafA1-treated WT cells, respectively (Figure 6A,B). In HKO cells, both mutants also accumulated MPL^+^ structures without enhancing MIL^+^ unsealed phagophores upon BafA1 treatment. However, unlike HaloTag-fused wild-type mATG8 (Figure 4A–G), the mean signal intensity of MPL-labeled mutant LC3B and GBRL2 markedly reduced in HKO cells compared to WT cells (Figure 6A,B). These results suggest that LIR-mATG8 LDS interactions play an important role in autophagosome biogenesis but are not required for autophagosome closure. Interestingly, a recent study indicates that mutations in the core LIR motif of the E2-like enzyme ATG3 affect LC3 lipidation [38]. We performed an *in vitro* mATG8 conjugation assay [39] and found that the LDS mutations attenuated lipid conjugation to form LC3B/GBRL2–PE (Fig. S5G and H). The inhibitory effect of LDS mutations on mATG8 lipidation was more pronounced with GBRL2 than LC3B, supporting the dominant role of GBR proteins in autophagosome biogenesis. Lastly, we conducted the HaloTag-GFP reporter processing assay to assess the impact of the LDS mutations on bulk autophagic flux. The mutations significantly reduced starvation-induced HaloTag processing in a BafA1-sensitive manner (Figure 6C–F). Collectively, these findings emphasize the significance of the LIR-mATG8 LDS interaction in ATG3-mediated mATG8 lipidation and autophagic flux, while autophagosome closure remains unaffected by disrupting this interaction.

## Discussion

The regulation of autophagosome formation by Atg8-family proteins in mammalian cells has been a subject of long-standing debate [8,40]. These proteins, along with their conjugation systems, reportedly play critical roles in various steps of autophagy, including phagophore formation and elongation [11–14], autophagosome closure and membrane integrity [15–17], fusion with lysosomes [18,19], and inner autophagosomal membrane degradation for functional autolysosome formation [20]. In this study, we investigated the role of mammalian ATG8s during autophagosome biogenesis by inhibiting autophagy at the autophagosome closure step through *VPS37A* depletion. Our findings provide clear evidence that mATG8s are essential for the initial stages of autophagosome formation, specifically phagophore initiation and expansion. Notably, the loss of mATG8 proteins significantly reduced the number and size of phagophores in *VPS37A* KO U-2 OS cells. Despite this reduction, sporadic small autophagic structures were still observed. These results align with yeast studies, where Atg8 deficiency similarly impedes phagophore formation, yet occasionally allows for the detection of small autophagosome-like structures [6,7,41]. Considering the nearly complete inhibition of autophagic flux observed in *mATG8* HKO U-2 OS cells, the small autophagosome-like structures formed in the absence of mATG8s may exhibit impaired lysosomal degradation efficiency, consistent with a previous finding in HeLa cells [18]. Thus, the differences in conclusions may stem from the brief duration of autophagosome formation [21] and the scarcity of early autophagic structures in electron micrographs under experimental conditions without autophagosome closure inhibition. Furthermore, the presence of mATG8 conjugation-independent alternative autophagy [42] in certain cell types may complicate data interpretation. Unlike *mATG8* HKO U-2 OS cells, which show nearly no autophagic flux, *mATG8* HKO HeLa cells exhibit a low level of starvation-induced bulk autophagic flux. This aligns with previous report that HeLa cells can undergo ATG7/12-independent noncanonical autophagy during nutrient starvation [43]. Future studies are needed to elucidate the mechanisms behind the residual bulk flux in *mATG8*-deficient HeLa cells during starvation and to understand why this activity is minimal in U-2 OS cells, with specific caution to cell-type specific effects and limitations when interpreting results.

Notably, we observed that autophagic structures accumulated in *GBR* TKO cells upon *VPS37A* loss were significantly smaller compared to *LC3* TKO and WT cells. This reduction in size, resulting from GBR depletion, coincided with impaired ULK1 recruitment. Consistently, phagophore-localized Atg1 has been shown to promote phagophore expansion in yeast [44]. Furthermore, while ATG8-mediated recruitment of Atg1/ULK1 is dispensable for triggering phagophore formation, the Atg1/ULK kinase complex remains essential for autophagosome formation [45,46]. Interestingly, our study also highlighted the impact of GBRL2 LDS mutation on lipidation, which was more pronounced than the effect of LC3B LDS mutation. Additionally, recent research suggests that ATG2 prefers interacting with GBR family proteins to promote phagophore expansion [14]. Therefore, the membrane growth defects observed upon GBR loss likely result from the higher affinity of LIR-containing ATGs to GBRs over LC3s [5,14]. Moreover, ectopic expression of LC3 family proteins restored starvation-induced autophagy in *mATG8* HKO cells to levels comparable with GBR overexpression. This finding suggests that the lower affinity of ATGs for the LC3 subfamily can be compensated by increasing their expression. Contrary to their distinct roles in membrane expansion, both LC3s and GBRs contribute to phagophore formation, indicating that LIR-LDS interactions are not crucial for this process. Analogous to yeast Atg8 [47], both LC3s and GBRs exhibit membrane tethering and fusion activities that are required for phagophore formation [48]. Notably, simultaneous knockout of both *LC3* and *GBR* subfamilies significantly hinders phagophore formation more than the loss of a single subfamily, suggesting that their membrane remodeling function may redundantly regulate this process.

Our study also sheds light on the relationship between mammalian ATG8s and ESCRT during autophagosome sealing. In *mATG8* HKO U-2 OS cells, VPS37A recruitment was severely impaired, but unlikely due to disruption of the VPS37A LIR-mATG8 LDS interactions reported in HeLa cells [17]. Neither deletion of the LIR-containing N-terminal unstructured region of VPS37A nor mutations in mATG8 LDSs significantly hindered autophagosome completion. Instead, our findings emphasize the crucial role of mATG8s in phagophore formation and growth. The absence of phagophore membranes likely causes the VPS37A targeting-defective phenotype in *mATG8* HKO U-2 OS cells, but LIR-independent ESCRT recruitment by mATG8s cannot be ruled out. The discrepancy regarding the requirement of the VPS37A LIR for autophagy between our study and the previous study [17] remains unclear. A recent biochemical analysis of the VPS37A N terminus highlighted the importance of its membrane curvature sensing function for ESCRT targeting to autophagosome formation sites [29]. Further investigations are warranted to fully understand the mechanisms of ESCRT targeting for autophagosome closure.

## Materials and methods

### Reagents

The following antibodies were used for western blotting (WB) and immunofluorescence (IF): actin (WB: MilliporeSigma, A5441; 1:10,000), CHMP4B (IF: ABclonal, A7402; 1:250), GABARAP (WB: Cell Signaling Technology, 13733S; 1:1,000), GABARAPL1 (WB: Abcam, ab86497; 1:1,000), GABARAPL2 (WB: Abcam, ab126607; 1:1,000), GFP-Booster Alexa Fluor 488 (IF: ChromoTek, gb2AF488; 1:1,000), HaloTag (WB: Promega Corporation, G928A; 1:1000), LC3A (WB: Cell Signaling Technology, 4599S; 1:1,000), LC3B (WB: Novus Biologicals, NB1002220; 1:5,000; coIP: MilliporeSigma, L7543; 2 µg antibody per 500 µg protein; Cell Signaling Technology, 83506S; 1:1,000), LC3C (WB: Cell Signaling Technology, 14736S; 1:1,000), SQSTM1/p62 (WB: PROGEN, GP62-C; 1:5,000; IF: 1,400), VPS37A (WB: MilliporeSigma, HPA024705; 1:2,500). The following secondary antibodies were used for WB and IF: Alexa Fluor 568 goat anti-rabbit IgG (IF: Invitrogen, A11036; 1:1,000), Alexa Fluor 647 goat anti-guinea pig IgG (IF: Invitrogen, A21450; 1:1,000), IRDye 680RD-conjugated donkey antibodies against mouse IgG (WB: LI-COR, 926–68072, 1:10000), guinea pig IgG (WB: LI-COR, 926–68077; 1:10000), rabbit (WB: LI-COR, 925–68073; 1:10,000); IRDye 800CW-conjugated donkey antibodies against rabbit IgG (WB: LI-COR, 926–32213; 1:10,000). ON-TARGETplus SMARTpool *nontargeting* (*NT*) (Dharmacon, D-001810-10-20) and *CHMP2A* (Dharmacon, L-020247-01-0020) were purchased from Horizon Discovery. The following plasmids were obtained through Addgene: epiCRISPR (135960; deposited by Yongming Wang) [49], FUGW-PK-*hLC3* (61460; deposited by Isei Tanida) [50], lentiCRISPRv2 (52961; deposited by Feng Zhang) [51], pMRX-IB-*HaloTag7-mGFP* (184903; deposited by Noboru Mizushima) [32], pMXs-IP-*EGFP-ULK1* (38193; deposited by Noboru Mizushima) [52], pMX-*STAT5A*-DN(−351)-*Neo* (83249; deposited by Andrew Brooks#). Multiplexed epiCRISPR constructs targeting *LC3s* (epiRISPR-*LC3s*) and *GBRs* (epiCRISPR-*GBRs*) were generated by tandemly inserting three gRNA expression unites (*RNU6* promoter – gRNA) targeting either subfamily into epiCRISPR through Gibson assembly. The targeting sequences are as follows: for *LC3* subfamily, *LC3A*: TCAAGATCATCCGGTGCG, *LC3B*: ATCCAACCAAAATCCCGGT, *LC3C*: CACTCTTGACAGGTGGTAG; for *GBR* subfamily, *GBR*: GATCTTCTCGCCCTCAGAG, *GBRL1*: ATTACCAGTAAGGTCAG, *GBRL2*: CGACAGGGTTCCGGTGAG. lentiCRISPRv2-*VPS37A* gRNA was generated as previously described [24]. For Figure 2A, pCDH1-CMV-*GFP-VPS37A*-EF1-*Neomycin* was generated by replacing the puromycin resistance gene (SalI – NotI) in pCDH1-CMV-*GFP-VPS37A*-EF1-*Puromycin* [24] to the neomycin resistance gene from pMX-*STAT5A*-DN(−351)-*Neo*. For Figure 2D, pCDH1-CMV-*GFP-VPS37A(Δ1–20)*-EF1-*Puromycin* was generated by Gibson assembly using pCDH1-CMV-*GFP-VPS37A*-EF1-*Puromycin* [24] as a template. The cDNAs encoding mammalian *ATG8s* (*LC3A, LC3B, LC3C, GBR, GBRL1, GBRL2*) were gifted from Felix Randow [53]. pCDH1-CMV-*HaloTag-mATG8* (*LC3A, LC3C, GBR, GBRL1, GBRL2*)-SV40-*Hygro* constructs were generated by replacing the *LC3B* cDNA with the cDNAs of *LC3A, LC3C, GBR, GBRL1* or *GBRL2* in the NheI – EcoRI site of the pCDH1-CMV-*HaloTag-LC3B*-SV40-*Hygro* vector [24]. pCDH1-CMV-*mATG8* (non-tagged)-EF1-*Puro* constructs were generated by subcloning individual genes encoding mATG8 members into the NheI – EcoRI site of the pCDH1-CMV-SV40-*Puro* vector (System Biosciences, CD510A–1). pCDH1-CMV-*Mut mATG8* (non-tagged)-EF1-*Puro* were generated by Gibson assembly using the following primer sets: for *LC3*^*K51A*^, *primer set 1*: 5’-CATAGAAGATTCTAGAGCTAGATGCCGTCGGAGAAGAC-3’, 5’-TCAGGTACAAGGAACGCTGTTTTATCCAGAACAGGAAGCTG-3’; *primer set 2*: 5’- GTTCTGGATAAAACAGCGTTCCTTGTACCTGACCATGTCA-3’, 5’- CGGATCCATTTAAATTCGTTACACTGACAATTTCATCCCGAA-3’. For *GBRL2*^*Y49A,L50A*^, *primer set 1*: 5’-CTCCATAGAAGATTCTAGAGCTAGATGAAGTGGATGTTCAAGGAGG-3’, 5’-GTGATATCAGATGGAACCGCCGCCTTCCGTTTGTCAATGTCAACAATCT-3’; *primer set 2*: 5’- GACATTGACAAACGGAAGGCGGCGGTTCCATCTGATATCACTGTGG-3’, 5’- CGGCCGCGGATCCATTTAAATTCGTCAGAAGCCAAAAGTGTTCTCT-3’. FUGW-*HaloTag-Mut mATG8* was generated by subcloning the respective cDNAs into the NheI – EcoRI site of the FUGW vector. pET28a-*his-LC3B Δ121–125*, pET28a-*his-ATG3* and pFast-BacI-*his6-mATG7* vectors were generated as previously described [39]. pET28a-*his-MutLC3B (K51A, Δ121–125)*, pET28a-*his-GBRL2 Δ117* and pET28a-*his-MutGBRL2 (Y49A, L50A, Δ117)* were generated by subcloning the respective cDNAs into the pET28a vector at the BamHI – XhoI site, incorporating a His-tag and thrombin cleavage site-encoding sequences at the 5’ end. All other reagents and materials were obtained from the following sources: 0.45 µm acrodisc sterile syringe filters (Cytiva, 4614), 1X antibiotic antimycotic solution (Cytiva, SV30079.01), bafilomycin A_1_ (Fisher Scientific, AAJ61835MCR), bovine serum albumin (MilliporeSigma 126,575), Capturem IP & CoIP Kit (TaKaRa Bio 635,721), cOmplete Mini Protease Inhibitor cocktail (MilliporeSigma 11,836,153,001), DMEM (Corning, 17–205-CV), amino acids-free DMEM (starvation medium; Wako Chemicals, 048–33575), fetal bovine serum (FBS; Avantor 97,068–091), GFP-Trap Magnetic Agarose (ChromoTek, gtma-20), 1X Glutamax (Fisher Scientific 35,050,061), HEPES (MilliporeSigma 83,264), IGEPAL CA 630 (MilliporeSigma 18,896), JetPrime (Polyplus-transfection 89,129), Lab-Tek II 8-well-chambered coverglasses (Fisher Scientific 155,409), Lipofectamine RNAiMAX Transfection reagent (Fisher Scientific 13,778,150), Magne HaloTag Beads (Promega Corporation, G728A), Mammalian Lysis Buffer (Promega Corporation, G9381), McCoy’s 5A (Corning, 10–050-CV), phosphate-buffered saline (PBS; Corning, 21–040-CV), paraformaldehyde (Fisher Scientific 15,710), protease inhibitor cocktail (MilliporeSigma, P0044), 50X protease inhibitor cocktail (Promega Corporation, G6521), Triton X-100 (MilliporeSigma, T8532).

### Cell culture and viral gene transduction

U-2 OS cells (ATCC, HTB-96) were cultured in McCoy’s 5A medium (Corning, 10–050-CV) supplemented with 10% FBS and 1X antibiotic antimycotic solution. *LC3* TKO, *GBR* TKO and *mATG8* HKO HeLa cells were gifted from the Lazarou lab (Walter and Eliza Hall Institute of Medical Research) [18] and maintained in DMEM media supplemented with 10% FBS, 1X antibiotic antimycotic solution, 25 mm HEPES, and 1X Glutamax. 293T/17 (ATCC, CRL-11268) cells were maintained in DMEM supplemented with 10% FBS and 1X antibiotic antimycotic solution. All cells were incubated at 37°C and 5% CO_2_. Lentivirus-mediated and retrovirus-mediated gene transduction were performed as previously described [24], using the Invitrogen ViraPower Lentiviral Expression System and the Phoenix Amphotropic Packaging System from the Nolan Lab at Stanford University, respectively.

### CRISPR/Cas9-mediated gene editing and siRNA-mediated gene silencing

To generate *LC3* TKO, *GBR* TKO and *mATG8* HKO U-2 OS cells, cells were transfected with either epiCRISPR-*LC3s*, epiCRISPR-*GBRs*, or both constructs using the jetPRIME transfection reagent for 1 day and selected with 2 µg/mL puromycin for 7 days. Single clones were then isolated by fluorescent activated cell sorting (FACS). After verification of the targeted gene knockouts, 3 clones from each group were pooled and used for experiments. For *VPS37A* knockouts, the lentiCRISPRv2 system was used as previously described [24]. siRNA-mediated gene silencing was performed using the Invitrogen Lipofectamine RNAiMAX Transfection Reagent according to the manufacture’s protocol.

### Western blotting

Total cell lysates were prepared in radio-immunoprecipitation assay buffer (150 mm NaCl, 25 mm Tris-HCl, pH 8.0, 0.1% sodium dodecyl sulfate [SDS], 1% Nonidet *p*-40 [NP-40/IGEPAL CA-630; MilliporeSigma, I8896], 0.5% deoxycholate [MilliporeSigma, D6750], 1 mm EDTA, pH 8.0) containing phosphatase inhibitor cocktail (MilliporeSigma, P5726, P0044) and subjected to SDS-PAGE followed by immunoblotting with the indicated antibodies. Signals were acquired and analyzed using an Odyssey CLx Imager (LICORbio) and LI-COR Image Studio software (version 5.2), respectively.

### Co-immunoprecipitation

For GFP and HaloTag immunoprecipitation (IP), whole cell lysates (WCLs) were prepared in the lysis buffer indicated below and subjected to immunoprecipitation using GFP-Trap beads or Magne HaloTag beads. After overnight incubation with WCLs, the beads were transferred to a new tube, washed five times with the following wash buffer, and then analyzed by western blotting. For GFP IP: 0.5% NP-40 lysis buffer (10 mm Tris-HCl, pH 7.4, 150 mm NaCl, 0.5 mm EDTA, 0.5% NP-40), 0.05% NP-40 wash buffer (10 mm Tris-HCl, pH 7.5, 150 mm NaCl, 0.5 mm EDTA, 0.05% NP-40). For HaloTag IP: Promega Corporation Mammalian Lysis Buffer containing a protease inhibitor cocktail, 0.005% NP-40 wash buffer (PBS, 0.005% NP-40). LC3B IP was conducted using an anti-LC3 antibody (MilliporeSigma, L7543) and the Takara Capturem IP & Co-IP Kit, following the manufacturer’s protocol, and analyzed by western blotting.

### Immunofluorescence and confocal microscopy

For immunofluorescence, cells were grown overnight on Nunc Lab-Tek II Chambered Coverglass (Fisher Scientific, 12-565-470), fixed in 4% paraformaldehyde-Dulbecco’s phosphate-buffered saline (DPBS; Corning, 21–030-CV) for 10 min, permeabilized with 0.25% Triton X-100-PBS for 5 min, blocked in 10% normal goat serum (SouthernBiotech, 0060–01)-PBS for 1 h, and incubated with the primary antibody overnight at 4°C followed by the secondary antibody for 1 h at room temperature (RT). For the detection of GFP-ULK1 signals, ChromoTek GFP-Booster Alexa Fluor 488 was used to enhance GFP signals. Images were acquired on an inverted Leica SP8 FALCON point scanning confocal microscope with a Leica 63×/1.2NA HC Plan Apo water immersion objective lens using Leica’s LAS X acquisition software (version 3.5.7.23225) and a pinhole size of 111.4 µm (1 A.U. for 580 nm emission). Experiments with multiple fluorophores used sequential acquisition. A 200 hz galvo speed with 4× line averaging and confocal zoom of 1 led to images with dimensions of 4096 × 4096 and 45 nm pixel sizes. Huygens Professional v24.04 (Scientific Volume Imaging, The Netherlands) was used to deconvolve all images.ilastik [54] was used for pixel classification. Manual selection of cells with similar expression levels for analysis was done through Fiji using brightfield channels for identifying cell boundaries. Batch analysis relied on custom ImageJ macros. Imaris (Bitplane) software was used for visualization.

### Autophagy assays

A HaloTag-based reporter processing assay was conducted as described previously [32]. Briefly, cells stably expressing HaloTag-GFP were incubated with tetramethylrhodamine (TMR)-conjugated, membrane-permeable HT ligand (MPL) in complete culture medium at a 1:25,000 ratio at 37°C for 20 min. After rinsing twice with DPBS, cells were either immediately harvested or further incubated in starvation medium with or without 100 nM BafA1 for 6 h and subjected to western blot analysis. A HaloTag-LC3 autophagosome completion assay was performed as described previously [25]. In brief, cells stably expressing HaloTag-mATG8 were incubated in 1× MAS buffer (220 mm mannitol [Fisher Scientific, M120–500], 70 mm sucrose [Fisher Scientific 217,610], 10 mm KH_2_PO_4_, 5 mMMgCl_2_, 2 mm HEPES 1 mm EGTA, pH 7.4) containing 3 nM XF Plasma Membrane Permeabilizer (Seahorse Bioscience 102,504–100) and 3.5 μM Alexa Fluor 488-conjugated, membrane-impermeable HT ligand (MIL) at 37°C for 15 min, fixed in 4% PFA for 5 min, incubated with 5 μM TMR for 30 min at RT, and analyzed by confocal microscopy.

### Electron microscopy

U-2 OS cells were grown overnight on 60-mm Permanox dishes (Electron Microscopy Sciences 70,350), incubated in starvation medium for 3 h, fixed in 2.5% glutaraldehyde, 2% paraformaldehyde in 0.1 M phosphate buffer (pH 7.4) for 1 h at RT followed by postfixation buffer (1% osmium tetroxide, a1.5% potassium ferrocyanide in 0.1 M phosphate buffer, pH 7.4) for 30 min at RT, dehydrated in a graduated ethanol series, acetone, and embedded in LX-112 (Ladd Research, Williston, VT). Thin sections (65 nm) were stained with uranyl acetate and lead citrate and viewed in a JEOL JEM1400 Transmission Electron Microscope (JEOL USA Inc., Peabody, MA, USA) located at the Penn State College of Medicine TEM Facility (RRID Number: SCR_021200).

### Protein expression and purification

ATG3, LC3B (Δ121–125), GBRL2 (Δ117) and their LDS mutant proteins were purified as described previously [39,55]. In brief, plasmids containing target genes were transformed into chemically competent Rosetta (DE3) pLysS cells (SIGMA 70,956-M) for expression. A single colony was selected to grow in a small volume of LB medium overnight at 37°C as a starter culture and then inoculated into a large volume of LB medium at 37°C until OD_600_ reached 0.6–0.8. The temperature was then lowered to 25°C and cells were induced with 0.5 mm IPTG (TEKNOVA 13,325) for ~16 h and then harvested by centrifugation. Mouse ATG7 (MmATG7) proteins were expressed using High Five insect cells as described previously [39,55]. Cell pellets containing expressed proteins were suspended in a lysis buffer of 20 mm phosphate (for ATG3 and mATG8s) or 50 mm HEPES containing 1 mm MgCl_2_ and benzonase nuclease (Millipore 70,746; for MmATG7), pH 7.5, 300 mm NaCl, 2 mm beta-mercaptoethanol (BME), and complete protease inhibitor cocktail (Roche 11,836,170,001). The cells were lysed by sonication on ice with 2 s on and 7 s off intervals for 18 min total duration. Cell debris was removed by centrifugation (Sorvall RC5B Plus Refrigerated Centrifuge) at 26,900 × g at 10°C for 30 min. Supernatants were collected and loaded onto a Ni-NTA column (HisTrap HP; Cytiva 17,524,801). The column was washed with PBS buffers (20 mm phosphate, pH 7.5, 300 mm NaCl, 2 mm BME) without and with 20 mm imidazole (Sigma, 288-32-4), and then eluted with PBS buffer containing 500 mm imidazole. The eluates were concentrated and further purified by size-exclusion chromatography (HiLoad 16/60 Superdex 200 or 75; Cytiva, 28-9893-35 or 28-9893 -34) using a buffer of 50 mm HEPES, pH 7.5, 1 M NaCl, and 1 mm DTT. Purified proteins were exchanged into a buffer of 50 mm HEPES, pH 7.5, 150 mm NaCl, and 2 mm TCEP (tris(2-carboxyethyl) phosphine; Sigma 51,805-45-9) for functional assays. Protein concentration was assayed using a Nanodrop (Thermo Scientific, Waltham, MA, USA).

### Liposomes preparation

Liposomes (4 mm lipids) were prepared in double-distilled H_2_O (ddH_2_O) by sonication [39,55]. Briefly, POPC (Avanti Polar Lipids 850,457), DOPG (Avanti Polar Lipids 840,475), and DOPE (Avanti Polar Lipids 850,725) in chloroform were added to a glass tube in corresponding molar ratio and dried to a thin film by spinning with heat for half an hour using a condenser rotor SpeedVac, followed by lyophilization overnight once the volatile organics were removed. Lipids were rehydrated with ddH_2_O for 1 h at 42°C, with a vortex and then frozen at − 80°C every 15 min. The rehydrated lipids were then for bath sonication (BRANSON 3510 R-MT Bransonic Ultrasonic Cleaner). The lipids were sonicated for 4 × 15 min intervals until clear and stored at room temperature for use.

### In vitro conjugation assay

*In vitro* LC3B – PE conjugation was conducted as previously described [39,55]. Typically, 5 μM LC3B, or GBRL2, or their mutants were mixed with 5.0 μM HsATG3, 0.5 μM MmATG7, 1 mm MgCl_2_ in a reaction buffer (50 mm HEPES, 150 mm NaCl, 2 mm TCEP, and pH 7.5) of 37 μL. 3.7 μL were used as a control without ATP. Then 1 mm ATP (0.45 μL of 100 mm ATP; Sigma 34,369-07-8) was added and the reaction was allowed to proceed for 30 min at 37°C for intermediate formation. 3.75 μL were removed to check for intermediate formations, and an additional 3.75 μL were incubated separately as a non-liposome containing control (CL). Liposomes (1 mm; 8.75 μL of 4 mm stock) were then added to the reaction system (final volume of 35 μL). The reaction proceeded at 37°C, with 5 μL aliquots removed at 0, 15, 30, 60, 120, and 180 min. Samples were mixed with 4 μL 4X protein loading buffer (10% w:v SDS, 10% BME, 40% glycerol, 250 mm Tris-HCl, pH 6.8, and 0.4% w:v bromophenol blue dye), and stored at −20°C until analyzed by 18% polyacrylamide gel electrophoresis after heating for 3 min. 4X loading buffer without BME was used for the gel analysis of intermediates (SurePAGE 10% Bis-Tris; GenScript, M00666). The gels were imaged on a Bio-Rad Chemidock MP imager and analyzed using ImageJ.

### Statistical analysis

Statistical significance was determined using Graph Pad Prism 7.0. Threshold for statistical significance for each test was set at 95% confidence (*p* < 0.05).

## Supplementary Material

Bui et al_supplementary materials_R3.docx
