# Supplementary material for: Blocking autophagosome closure manifests the roles of mammalian Atg8-family proteins in phagophore formation and expansion during nutrient starvation
Source: Autophagy. 2024 Dec 18;21(5):1059–74. doi: 10.1080/15548627.2024.2443300 (PMC12013414; doi:10.1080/15548627.2024.2443300)
Supplement: Bui et al_supplementary materials_R3.docx [file KAUP_A_2443300_SM6707.docx]

**Supplementary Materials for**

**Blocking autophagosome closure manifests the roles of mammalian Atg8-family proteins in phagophore formation and expansion during nutrient starvation**

Van Bui^a^, Xinwen Liang^a^, Yansheng Ye^b^, William Giang^c^, Fang Tian^b^, Yoshinori Takahashi^a^, Hong-Gang Wang^a,d^

^a^Division of Pediatric Hematology and Oncology, Department of Pediatrics, The Pennsylvania State University College of Medicine, Hershey, PA, USA

^b^Department of Biochemistry and Molecular Biology, The Pennsylvania State University College of Medicine, Hershey, PA, USA

^c^Advanced Light Microscopy Core Facility, The Pennsylvania State University College of Medicine, Hershey, PA, USA

^d^Department of Pharmacology, The Pennsylvania State University College of Medicine, Hershey, PA, USA

**Contact**

Yoshinori Takahashi [ytakahashi@pennstatehealth.psu.edu](mailto:ytakahashi@pennstatehealth.psu.edu) Department of Pediatrics, The Pennsylvania State University College of Medicine, 500 University DR, Hershey, PA 17033, USA

Hong-Gang Wang [huw11@psu.edu](mailto:huw11@psu.edu) Department of Pediatrics, The Pennsylvania State University College of Medicine, 500 University DR, Hershey, PA 17033, USA


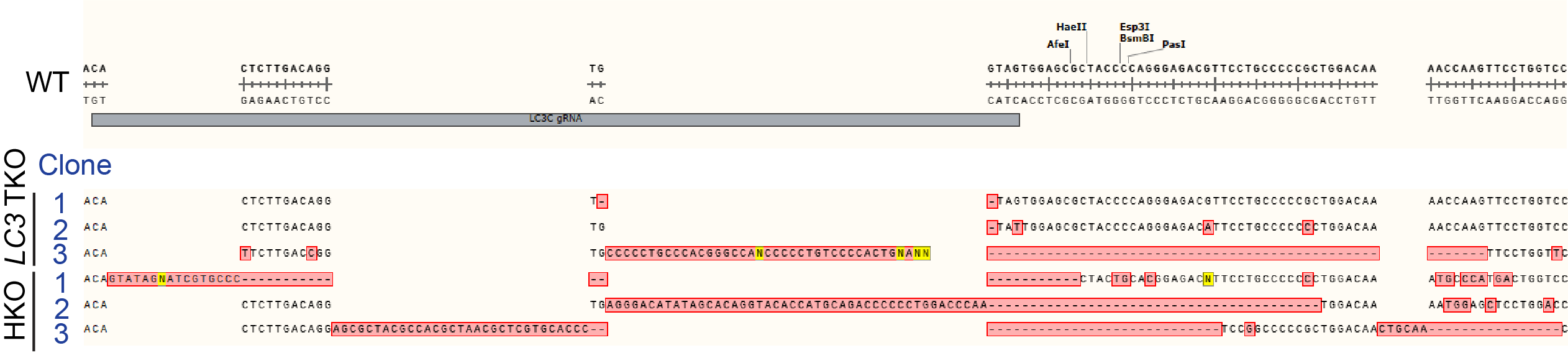


**Figure S1.** Confirmation of *LC3C* knockout in *LC3* TKO and HKO U-2OS cells. Sanger sequencing of *MAP1LC3C/LC3C* in *LC3* TKO and *mATG8* HKO U-2 OS cell clones. The *LC3C* gRNA targeting site, mutated regions, and unsequenced regions are highlighted in gray, red, and yellow respectively.


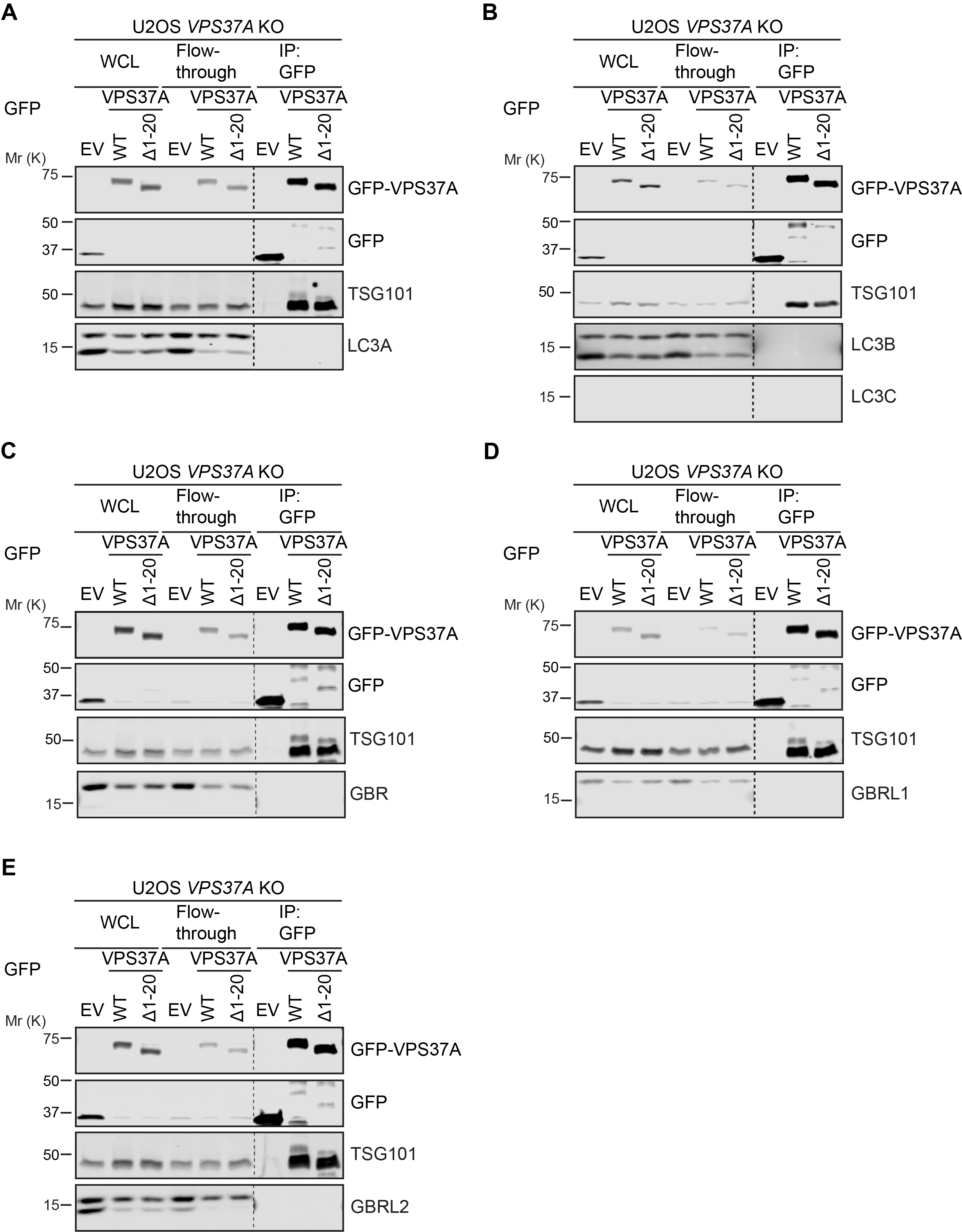


**Figure S2.** Both the wild-type and the Δ1-20 mutant forms of VPS37A form complexes with TSG101, but not with mATG8 family members in U-2 OS cells. Western blot analysis of whole cell lysates (WCL), anti-GFP immunoprecipitates (IP) and flow-through from the cells used for Figure 2D, using antibodies for GFP, TSG101, and either LC3A (**A**), LC3B (**B**), GBR (**C**), GBRL1 (**D**), or GBRL2 (**E**).


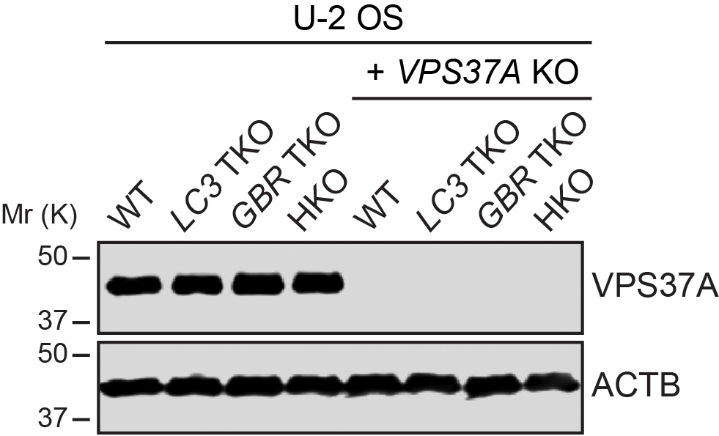


**Figure S3.** Validation of *VPS37A* knockout in WT, *LC3* TKO, *GBR* TKO, and HKO U-2 OS cells. Western blot analysis of the indicated U-2 OS cells.


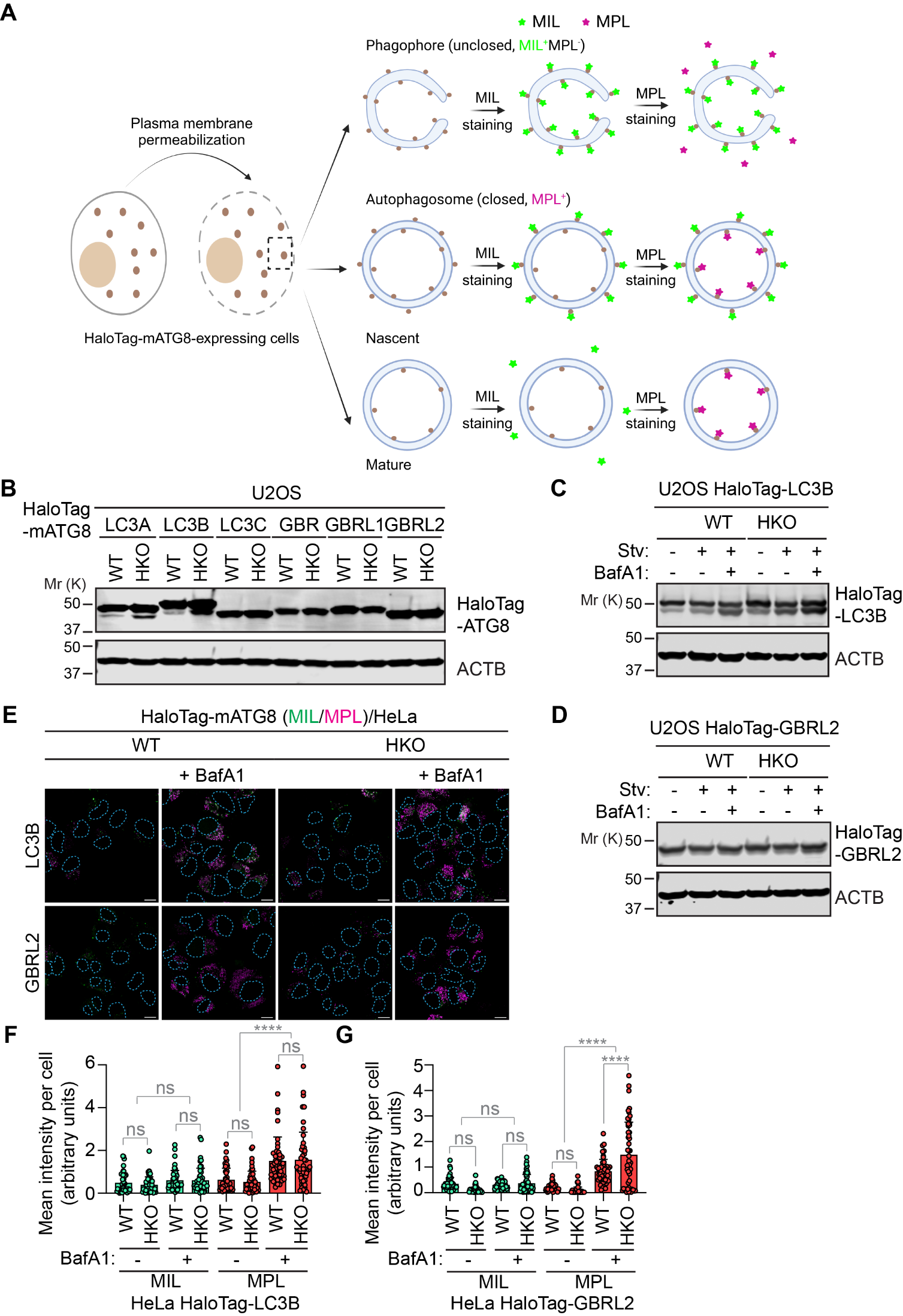


**Figure S4.** Overexpression of an individual mATG8-family member restores autophagy in HKO U-2 OS and HeLa cells. (**A**) Schematic diagram of the HaloTag-mATG8 autophagosome completion assay for monitoring the progression of autophagosome formation (Created in BioRender. Bui, V. (2024) BioRender.com/u84h344). (**B**) Western blot analysis of WT and HKO U-2OS expressing HaloTag-mATG8. (**C** **and** **D**) Western blot analysis of WT and HKO U-2 OS expressing HaloTag-LC3B or GBRL2 that were starved in the presence or absence of 100 nM BafA1 for 3 h. (**E**) Confocal images of the HaloTag-mATG8 autophagosome completion assay performed in WT and HKO HeLa cells expressing HaloTag-LC3B or -GBRL2. (**F and** **G**) Quantification of cytoplasmic fluorescence intensities of MIL and MPL in each cell in **E** (n = 50 cells). The mean fluorescence intensity, normalized to the cytoplasmic area, is shown. All values in **F** and **G** were presented as mean ± SD. One-way ANOVA test was performed followed by Tukey’s multiple comparison test. The *p*-values in **F**: ****p<0.0001; and in **G**: ****p<0.0001; ns, not significant.


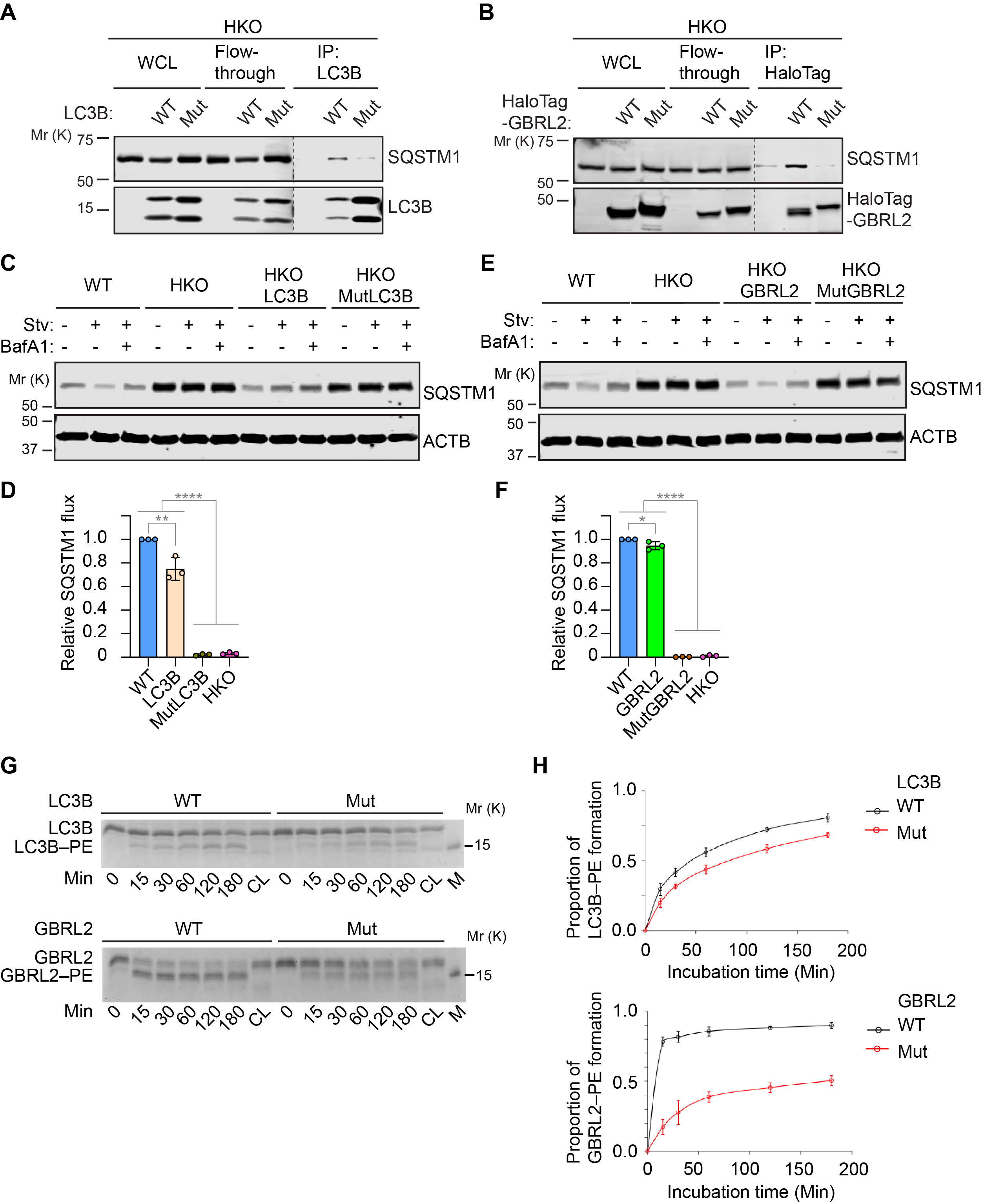


**Figure S5.** Mutations in the LIR docking sites (LDSs) of LC3B and GBRL2 attenuate their lipid conjugation in vitro. (**A and B**) Western blot analysis of WCL, flow-through and immunoprecipitates (IP) from the indicated HKO KO U-2 OS cells that were starved for 3 h. To prevent the autophagic degradation of SQSTM1 and LC3-II, cells were treated in the presence of 100 nM bafilomycin A_1_ (BafA1). (**C** **and** **E**) Western blot analysis of the indicated U-2 OS cells that were starved in the presence or absence of 100 nM BafA1 for 3 h. (**D** **and** **F**) Quantification of (‘Stv+BafA1’-’Stv’)/‘Stv+BafA1’ ratio relative to WT cells in **C** (**D**) and **E** (**F**) for starvation-induced SQSTM1 flux (n=3). (**G**) SDS-PAGE gel images of time-dependent phosphatidylethanolamine (PE) conjugation of recombinant LC3B, MutLC3B, GBRL2, and MutGBRL2 proteins. CL represents the control without liposomes at 180 min. (**H**) Plots of time-dependent PE conjugation of LC3B and GBRL2 in **G**. Quantification of conjugation reactions was obtained from three separate measurements (n=3). All values in **C**, **D**, and **H** are presented as mean ± SD. One-way ANOVA test was performed followed by Tukey’s multiple comparison test. The *p*-values in **D**: ****p<0.0001, **p=0.0011; and in **F**: ****p<0.0001, *p=0.0247.
